# Evaluation of trabecular bone structure using fractal analysis in patients with peri-implant health and disease

**DOI:** 10.1186/s12903-026-08714-8

**Published:** 2026-05-28

**Authors:** Okan Özen, Vesile Elif Toy, Duygu Çelik Özen

**Affiliations:** 1 Department of Periodontolgy, Faculty of Dentistry, Inonu University, Malatya, Turkey; 2 Department of Oral and Maxillofacial Radiology, Faculty of Dentistry, Inonu University, Malatya, Turkey

**Keywords:** Fractal analysis, Peri-implant health, Peri-implant mucositis, Peri-implantitis

## Abstract

**Background:**

Peri-implant diseases are associated with inflammatory changes affecting the peri-implant bone; however, objective radiographic markers for assessing trabecular bone structure are limited. Fractal dimension (FD) analysis is a noninvasive method for measuring trabecular complexity. This study aimed to assess peri-implant trabecular architecture using FD and to investigate its relationship with clinical parameters of peri-implant health and disease.

**Methods:**

Patients with dental implants were examined at a university periodontology clinic. Plaque index (PI), gingival index (GI), bleeding on probing (BOP), and probing depth (PD) were recorded at six sites for each implant. Implants were classified as having peri-implant health, mucositis, or peri-implantitis based on a combination of clinical and radiographic criteria. According to this classification, 75 mandibular implants were included in the study using panoramic images. Regions of interest (ROI) were determined to evaluate the trabecular bone in the mesial and distal vicinities of the implants, and mean FD values were calculated using standardized box-counting methods.

**Results:**

FD values progressively decreased from peri-implant health to mucositis and peri-implantitis (*p* < 0.01). FD showed a weak but statistically significant negative correlation with PD and BOP (*p* < 0.05), whereas correlations with PI and GI did not reach statistical significance. Peri-implantitis areas exhibited the lowest FD values and the highest inflammation load.

**Conclusions:**

Decreased trabecular bone complexity, as assessed by FD, was associated with peri-implant disease severity. FD can serve as a complementary radiographic marker in the assessment of peri-implant bone changes and can support the clinical evaluation of peri-implant disease in practice.

## Background

Implant-supported dental restorations have become a widely accepted and effective option for the rehabilitation of missing teeth. This widespread use has been accompanied by an increasing prevalence of peri-implant disease. Peri-implant mucositis and peri-implantitis are the most prevalent biological complications involving peri-implant tissues. Peri-implant mucositis is defined as inflammation of the soft tissues surrounding the implant without any associated bone loss, and it has been reported in approximately 80% of cases [1]. Clinically, bleeding on probing without accompanying radiographic bone loss is considered the hallmark of peri-implant mucositis. Although bleeding on probing is the primary clinical sign of this soft-tissue inflammation, additional findings may include erythema, swelling, and suppuration [2]. In contrast, if radiographic bone loss is detected in addition to bleeding on probing, a diagnosis of peri-implantitis is considered [3]. Peri-implantitis is a pathological condition characterized by inflammation of the peri-implant soft tissues, accompanied by progressive supporting bone loss that exceeds the physiological bone remodeling process.

Current evidence indicates that peri-implantitis typically develops within the first few years after the implant is functionally loaded and progresses at an accelerating rate [4]. At this stage, inflammatory changes remain confined to the peri-implant soft tissues; however, if left untreated, the condition may progress to irreversible marginal bone loss and is therefore regarded as a precursor of peri-implantitis [5, 6]. In summary, peri-implant mucositis is a reversible inflammatory condition confined to the peri-implant soft tissues, whereas peri-implantitis is a more advanced disease stage characterized by progressive loss of the supporting bone [7].

This increasing disease burden underscores the importance of early detection and accurate assessment of peri-implant tissue changes. This may impose a substantial burden on oral healthcare services in the future [8]. Given the importance of early diagnosis, fractal analysis offers a noninvasive radiographic approach for assessing the peri-implant bone structure. The fractal dimension (FD) quantifies the complexity and self-similarity of the trabecular structure, providing valuable information on bone heterogeneity beyond simple density measurements [9]. Fractal analysis allows for the reliable assessment of microarchitectural changes in the mandibular trabecular bone associated with systemic conditions (such as osteoporosis, diabetes, and chronic kidney disease), local pathologies (including periodontitis, cysts, post-endodontic changes, and bisphosphonate-related osteonecrosis), and surgical or implant-related procedures [10]. Among the imaging modalities used in fractal analysis, panoramic radiography is widely preferred in dentistry because of its low radiation dose and cost-effectiveness. Its ability to display all dentoalveolar structures in a single image has made it a common tool in routine clinical practice [11].

This study aimed to evaluate peri-implant bone on panoramic radiographs using fractal analysis in cases of peri-implant health, peri-implant mucositis, and peri-implantitis and to compare the FD values across these conditions.

## Methods

The study protocol was reviewed and approved by the Inonu University Scientific Research Ethics Committee (approval no. 2025/8121). The study was conducted using fully anonymized archived data in accordance with institutional and national regulations. All procedures were performed in compliance with the Declaration of Helsinki.

An a priori power analysis was performed prior to data collection using G*Power software (version 3.1.9.7; Heinrich-Heine-Universität Düsseldorf, Germany). The primary outcome variable used for the calculation was the total FD value. The analysis was based on a one-way ANOVA model with three independent groups. In the absence of pilot data specific to the present population and considering the variability of reported FD differences in the peri-implant literatüre [12–14], a medium effect size (f = 0.25) was assumed according to Cohen’s conventional classification for ANOVA models. This assumption reflects a clinically meaningful intergroup difference while avoiding overestimation of effect magnitude. With a significance level of α = 0.05 and a desired statistical power of 80% (1 − β = 0.80), the minimum required sample size was calculated as 75 implants (25 per group).

### Patient selection

**Fig. 1:**
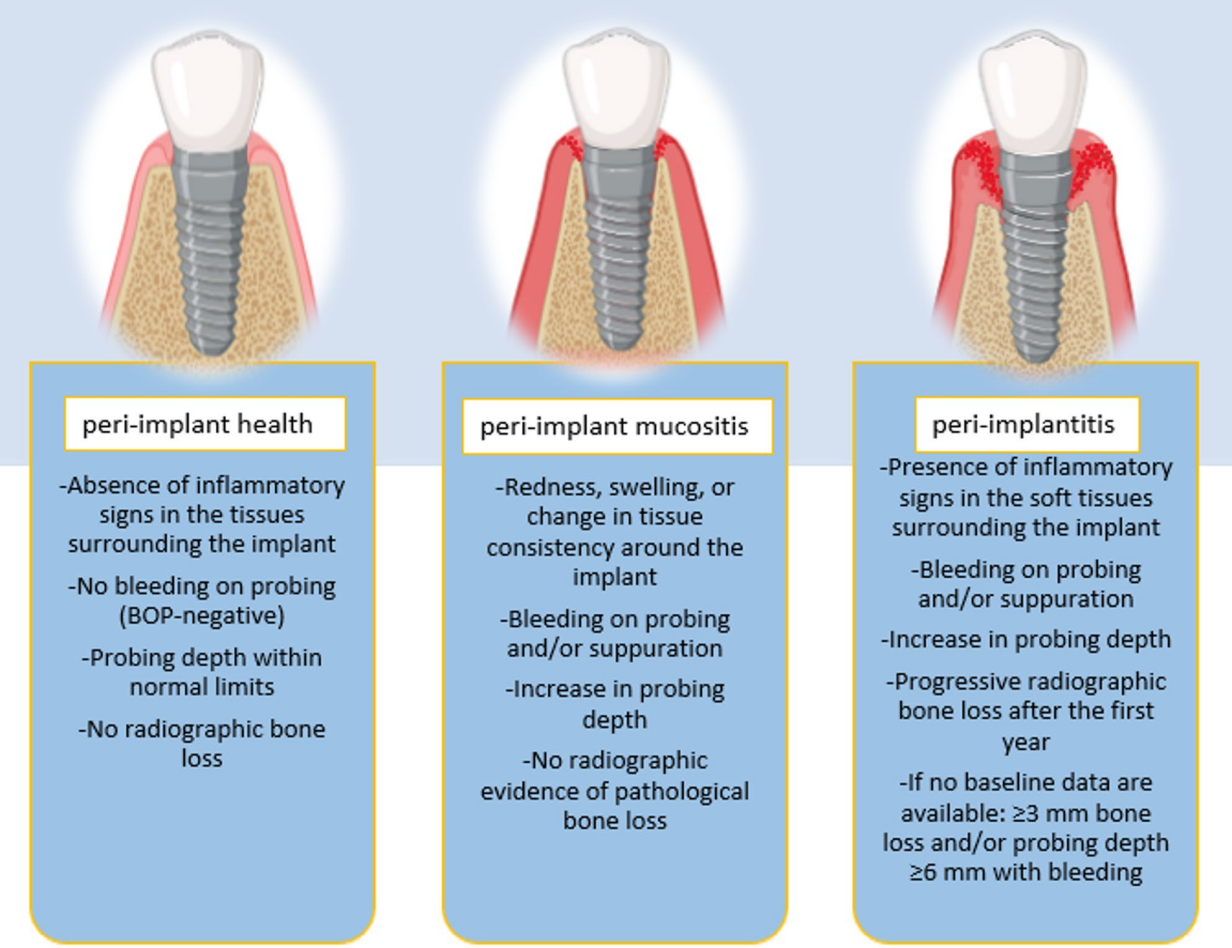
Clinical and radiographic criteria illustrating peri-implant health, peri-implant mucositis, and peri-implantitis

Patients who had previously received dental implant treatment and subsequently presented to the Periodontology Clinic of Inonu University Faculty of Dentistry were assessed by a periodontist (VET) for eligibility for inclusion in this study. The peri-implant health status of the participants was assessed according to the criteria of the 2017 World Workshop on the Classification of Periodontal and Peri-implant Diseases and Conditions [15]. The plaque index (PI) [16], gingival index (GI) [17], bleeding on probing (BOP) [18], and probing depth (PD) values were recorded from six sites (mid-buccal, mid-lingual, mesio-buccal, disto-buccal, disto-lingual, and mesio-lingual) around each implant using a Williams periodontal probe (Hu-Friedy, Chicago, IL, USA). The percentage of BOP was calculated as the proportion of bleeding sites to the total number of sites examined. Additionally, radiographic evaluation was performed to confirm the peri-implant bone levels. Based on these parameters, the patients were classified into peri-implant health, peri-implant mucositis, and peri-implantitis groups (Fig. 1).

### Inclusion criteria

1. The presence of one implant in the mandibular posterior region with a completed prosthetic restoration that had been functionally loaded for at least one year, diagnosis of healthy peri-implant mucosa or peri-implant mucositis or peri-implantitis.
2. Dental implants that had been functionally loaded for at least 12 months were included.
3. High-resolution panoramic radiographs allow clear visualization of the peri-implant area.
4. Systemically healthy individuals with no history of systemic diseases or medications that could affect bone metabolism.

### Exclusion criteria

1. Systemic conditions that may alter bone metabolism (e.g., osteoporosis, thyroid disorders, and metabolic bone diseases).
2. Systemic conditions known to adversely affect peri-implant tissue health (e.g., uncontrolled diabetes mellitus).
3. Regular use of medications that may influence bone metabolism (e.g., corticosteroids, bisphosphonates, and chemotherapeutic agents).
4. History of radiotherapy or chemotherapy in the head and neck region.
5. Signs of active periodontal disease or untreated periapical pathology.
6. Previous surgical interventions, grafting, or regenerative procedures at the relevant site.
7. Evidence of occlusal overload or prosthetic misfit.
8. Current smoking.
9. Insufficient clinical or radiographic records.

Based on combined clinical and radiographic examinations, 75 mandibular implants (25 peri-implant healthy, 25 peri-implant mucositis, and 25 peri-implantitis) from 60 patients were included. Peri-implant health was defined as the absence of bleeding on probing and radiographic bone loss, peri-implant mucositis as the presence of bleeding on probing without radiographic bone loss, and peri-implantitis as bleeding on probing accompanied by radiographic evidence of supporting bone loss. Panoramic radiographs of the implants were used for the fractal analysis.

#### Imaging protocol

All panoramic images were obtained using the same radiographic device (Planmeca Proline XC; Helsinki, Finland; exposure parameters: 66 kVp, 5 mA, 18 s). During image acquisition, the Frankfort horizontal plane was aligned parallel to the floor and the sagittal plane was aligned with the vertical reference line of the device. The images were recorded using the Romexis software and exported in TIFF format.

#### Fractal analysis

Fractal analysis was performed using ImageJ 1.54k, an image analysis software developed by the National Institutes of Health and freely available at “http://rsb.info.nih.gov”.

The images saved in TIFF format were opened in the ImageJ program by an oral and maxillofacial radiology specialist (DCO). Prior to measurements, image calibration was performed in ImageJ using the embedded scale reference on the panoramic images. Two rectangular regions of interest (ROIs) were selected from the mesial and distal peri-implant bone. To ensure standardization and reproducibility, a fixed-width ROI template (15 pixels; approximately 2.4 mm) was applied consistently to all images, aligned parallel to the long axis of the implant. The upper boundary of each ROI was consistently aligned with the implant shoulder, while the height was adjusted according to the implant length to encompass the maximum possible area of adjacent trabecular bone within the intraosseous portion. Care was taken to exclude the lamina dura, periodontal ligament, or root apices of adjacent teeth to avoid misleading results. To ensure intra-examiner reliability, a subset of radiographs (30%) was randomly re-evaluated by the same specialist (DCO) after a five-week interval, blinded to the initial measurements and clinical data; the consistency of these repeated measurements was then verified using the intraclass correlation coefficient (ICC). In cases of multiple implants within the same patient, each was included as a separate unit only if located in different quadrants or contralateral sides, ensuring that each ROI represented a distinct anatomical and trabecular environment.

**Fig. 2:**
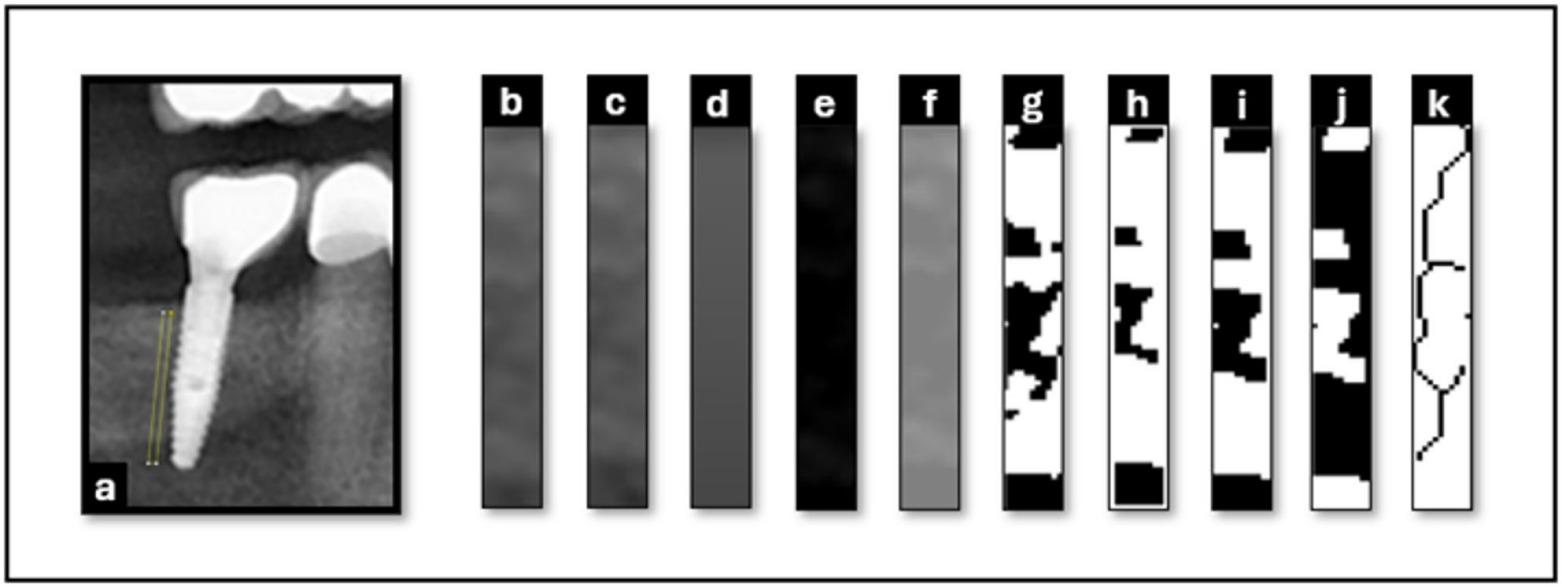
Fractal analysis workflow. **a** Selection of the ROI on the panoramic radiograph; (**b**) cropped and duplicated ROI image; (**c**) Gaussian-blurred version; (**d**) difference image obtained by subtracting the blurred image from the original; (**e**) normalized image created by adding a gray value of 128; (**f**) binarized image; (**g**) eroded image; (**h**) dilated image; (**i**) inverted image; and (**j**) final skeletonized image

The box-counting method described by White and Rudolph [19] was used for the fractal analysis. The ROIs were selected on the panoramic images, cropped, and saved in an 8-bit format. A Gaussian filter (σ = 35 pixels) was applied to the duplicated image to eliminate fine variations, and the resulting blurred image was then subtracted from the original image using the “subtraction” function. A gray value of 128 was added to each pixel, which was set as the threshold. After thresholding, the image was converted to binary format, an erosion step was applied to reduce noise, and a dilation step was performed to enhance the visibility of trabecular structures. The image was inverted to enhance the visibility of the trabecular structures. The image was subsequently processed using the skeletonization step to display the central lines of the trabecular structure as a single-pixel-wide pattern. Fractal analysis was then performed on the skeletonized images using the “box counting” function in ImageJ software. Square sizes of 2, 3, 4, 6, 8, 12, 16, 32, and 64 pixels were used in the analysis, and the number of squares at each size was calculated to generate a logarithmic plot. A best-fit linear regression line was obtained for the points on the graph, and the slope of this line was recorded as the FD value, which represents the complexity of the trabecular structure (Fig. 2).

### Statistical analysis

Data were analyzed using R software (version 4.4.1). The normality of the variables was assessed using the Kolmogorov–Smirnov and Shapiro–Wilk tests. As the FD values and clinical parameters did not follow a normal distribution, group comparisons were performed using the nonparametric Kruskal–Wallis H test. For variables showing significant differences, multiple comparisons were conducted using Dunn’s post hoc test, and the results are represented in the table using superscript letters. Effect sizes for the Kruskal–Wallis test were calculated using eta squared (η²). Differences in bleeding on probing among the groups were evaluated using the chi-square test, and the strength of association was reported using Cramér’s V. Differences in BOP among the groups were evaluated using the chi-square test. Receiver operating characteristic (ROC) analysis was performed to evaluate the diagnostic performance of total FD values in distinguishing between the study groups, and the area under the curve (AUC) was calculated. The relationship between total FD values and clinical parameters was evaluated using Spearman’s correlation analysis. Intra-observer agreement for FD measurements was assessed using the intraclass correlation coefficient (ICC). A significance level of *p* < 0.05 was considered statistically significant.

## Results

The study included 75 mandibular implants placed in patients aged between 22 and 72 years (mean age: 50.33 ± 10.94 years), comprising 27 women and 33 men. There were no statistically significant differences among the groups in terms of age (Group PH: 48.28 ± 11.07; Group PM: 49.08 ± 12.25; Group PI: 54.24 ± 7.71) (Table 1). The distribution of the implant sites included in the study is presented in Table 2.

**Table 1:** Distribution of demographic variables according to the groups

Parameter	Peri-implant health	Peri-implant mucositis	Peri-implantitis	*p*
Age (years)	48.28 ± 11.07	49.08 ± 12.25	54.24 ± 7.71	0.125*
Sex (Male/Female)	9 / 16	15 / 10	12 / 13	0.236**

* Kruskal–Wallis H test

** Chi-square test; groups sharing the same superscript letter do not differ significantly

**Table 2:** Distribution of implant sites

Site	Peri-implant health *n* (%)	Peri-implant mucositis *n* (%)	Peri-implantitis *n* (%)
34	1 (4.0)	2 (8.0)	2 (8.0)
35	2 (8.0)	3 (12.0)	1 (4.0)
36	7 (28.0)	5 (20.0)	3 (12.0)
37	2 (8.0)	1 (4.0)	7 (28.0)
44	2 (8.0)	1 (4.0)	2 (8.0)
45	2 (8.0)	3 (12.0)	2 (8.0)
46	6 (24.0)	6 (24.0)	5 (20.0)
47	3 (12.0)	4 (16.0)	3 (12.0)

### Clinical parameters

The clinical periodontal parameters are summarized in Table 3. PD differed significantly among the groups (*p* < 0.001), with the highest values observed in the peri-implantitis group, demonstrating a large effect size (η²=0.747). GI values were comparable between the peri-implant mucositis and peri-implantitis groups, whereas both groups exhibited significantly higher values than the peri-implant health group (*p* < 0.001; η²=0.34). Similarly, PI values were higher in the peri-implant mucositis and peri-implantitis groups than in the healthy group, with a significant difference observed between peri-implant health and peri-implantitis (*p* = 0.005; η²=0.11**)**. BOP was infrequent in the peri-implant health group but was present in all implants diagnosed with peri-implant mucositis or peri-implantitis. Accordingly, the percentage of BOP was significantly higher in the peri-implant mucositis and peri-implantitis groups than in the peri-implant health group (*p* < 0.001), with a very strong association (Cramér’s V = 0.97).

**Table 3:** Comparison of clinical parameters among peri-implant health, peri-implant mucositis, and peri-implantitis groups

Parameter	Peri-implant health	Peri-implant mucositis	Peri-implantitis	Test statistic	*p*-value	ƞ2 V
PD (mm)	2.00 (1.20–2.66)^a^	2.5 (1.70–3.83) ^b^	5.66 (4.00-8.66)^c^	H = 57.28	< 0.001^x^	ƞ2 = 0,747
GI	1 (0–2)^a^	2 (1–2)^b^	2 (1–2)^b^	H = 27.38	< 0.001^x^	ƞ2 = 0,34
PI	0 (0–2)^a^	1 (0–2)^ab^	1 (0–2)^b^	H = 10.48	0.005^x^	ƞ2 = 0,11
BOP (%)	4%^a^	100%^b^	100%^b^	χ²=70.59	< 0.001^xx^	V = 0,97

^*x*^ *Kruskall Wallis H Test*

*xx Chi-square test*

*a-b-c there is no difference between groups with the same letter*

*ƞ2 = Eta squared V= Cramer’s V*

### Fractal analysis

**Fig. 3:**
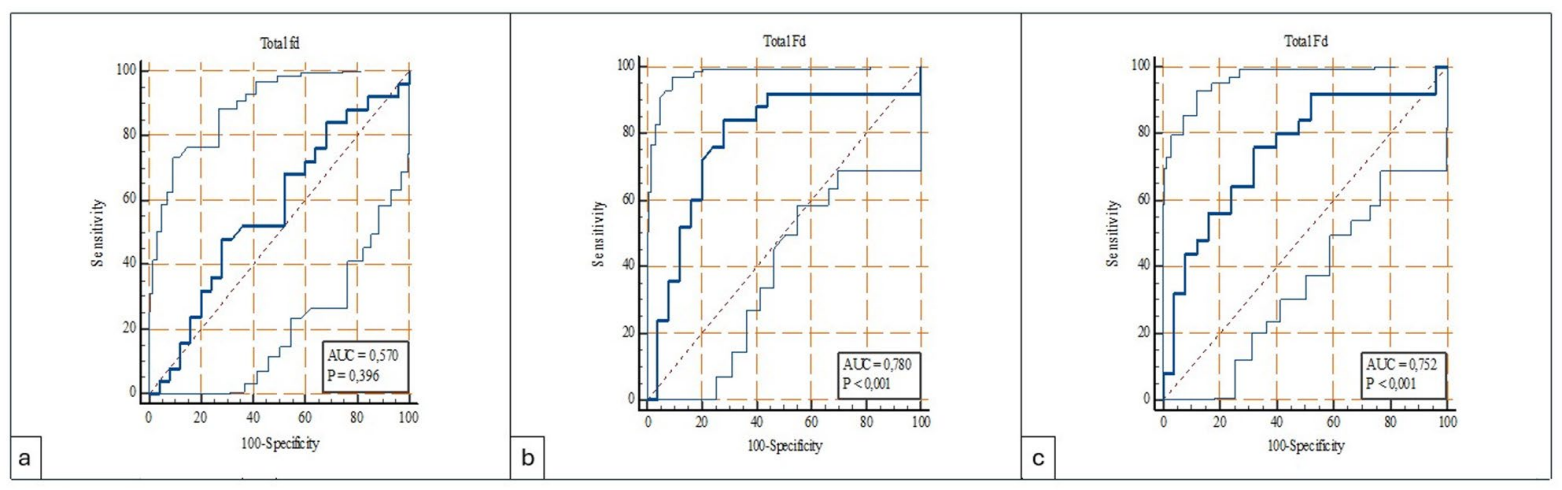
ROC curves showing the diagnostic performance of total FD values for differentiating (**a**) peri-implant health vs peri-implant mucositis, (**b**) peri-implant health vs peri-implantitis, and (**c**) peri-implant mucositis vs peri-implantitis

Intra-observer agreement for categorical radiographic bone changes demonstrated substantial agreement (κ = 0.84). For continuous FD measurements, ICC analysis indicated high reliability (ICC = 0.90; 95% CI [0.83–0.94]). A statistically significant difference was observed among the groups in terms of the median mesial FD values (*p* = 0.004; η²=0.117). Although no significant difference was observed between the healthy and peri-implant mucositis groups, the FD values in the peri-implantitis group were significantly lower than those in both the healthy and peri-implant mucositis groups. When distal FD scores were evaluated, no significant difference was observed between the healthy and peri-implant mucositis groups; however, the peri-implantitis group exhibited significantly lower scores than the other two groups (*p* = 0.002; η²=0.136). Although no statistically significant difference was observed between the healthy and peri-implant mucositis groups, significant differences were observed between the healthy and peri-implantitis groups and between the peri-implant mucositis and peri-implantitis groups. A statistically significant difference was observed in the total FD values among the groups (*p* < 0.001; η² 0.168). The total FD values were lower in the peri-implantitis group (0.985) than in the peri-implant health (1.092) and peri-implant mucositis (1.055) groups. No significant difference was observed between the healthy and peri-implant mucositis groups; however, significant differences were found between the healthy and peri-implantitis groups and between the peri-implant mucositis and peri-implantitis groups (Table 4). ROC analysis demonstrated that total FD showed no discriminatory ability between peri-implant health and mucositis (AUC = 0.570), whereas moderate diagnostic performance was observed for distinguishing peri-implantitis from peri-implant health (AUC = 0.780,) and peri-implant mucositis (AUC = 0.752,) (Fig. 3).

**Table 4:** Comparison of FD values among peri-implant health, mucositis, and peri-implantitis groups

	Peri-implant health	Peri-implant mucositis	Peri-implantitis	Test Statistic	*p*	ƞ2
Mesial FD	1.089 (0.814–1.538)^a^	1.055 (0.940–1.507)^a^	1.008 (0.799–1.500)^b^	H = 10.66	0.004	0,117
Distal FD	1.102 (0.796–1.526)^a^	1.087 (0.841–1.511)^a^	1.011 (0.813–1.490)^b^	H = 12.03	0.002	0,136
Total FD	1.092 (0.805–1.426)^a^	1.055 (0.894–1.509)^a^	0.985 (0.810–1.494)^b^	H = 14.40	*p* < 0.001	0,168

^*x*^ *Kruskall Wallis H Test*

^*a-b*^ *there is no difference between groups with the same letter*

*ƞ2  =Eta squared*

The correlations between FD values and clinical parameters are shown in Table 5. Spearman’s correlation analysis demonstrated a weak but statistically significant negative correlation between total FD and PD (*p* = − 0.391, *p* = 0.001). A weak negative correlation was also observed between total FD and BOP (*p* = − 0.279, *p* = 0.016), which was statistically significant. Although negative correlations were similarly detected between total FD and GI and PI, these associations were not statistically significant. (*p* > 0.05).

**Table 5:** Spearman correlation coefficients between total FD and clinical periodontal parameters

		Total FD	BOP	PD	GI	PI
Total FD	rho	1,000				
	p-value					
BOP	rho	-0,279	1,000			
	p-value	0,016^*^				
PD	rho	-0,391	0,602	1,000		
	p-value	0,001^**^	0,001^**^			
GI	rho	-0,206	0,641	0,382	1,000	
	p-value	0,077	0,001^**^	0,001^**^		
PI	rho	-0,194	0,336	0,423	0,346	1,000
	p-value	0,095	0,003^**^	0,001^**^	0,002^**^	

^*^P < 0,*05*

^**^P < 0,*01*

*Rho= Spearman’s ρ*

## Discussion

Although clinical parameters remain the primary determinants for evaluating peri-implant tissue health, recent advances in quantitative assessment of bone microarchitecture have enabled a more comprehensive understanding of disease progression [10]. Combining clinical findings with tissue-level alterations may provide a multidimensional perspective on the transition from peri-implant health to peri-implantitis.

In the present study, FD values progressively decreased from peri-implant health to peri-implant mucositis and peri-implantitis, with the most pronounced reduction observed in peri-implantitis cases. These findings suggest that increasing inflammation and tissue destruction are accompanied by progressive irregularity of the trabecular bone architecture. Fractal analysis enabled quantitative characterization of these structural alterations and may reflect microarchitectural changes associated with disease progression. However, because panoramic radiographs provide two-dimensional projections, the observed FD alterations should be interpreted as projected trabecular pattern changes rather than direct three-dimensional microarchitectural measurements.

Previous studies have characterized peri-implant disease progression using multidimensional clinical parameters. Heitz-Mayfield et al. [3] and Ramberg et al. [20] reported that peri-implant mucositis is primarily associated with plaque-induced superficial inflammation characterized by increased GI and BOP values, whereas PD generally remains relatively stable. Similarly, Porras et al. [21] suggested that peri-implant disease begins with plaque accumulation and progresses through increasing inflammatory changes before advancing to a destructive phase associated with increased PD. In contrast, peri-implantitis has been described as a condition characterized by increased PD, generalized BOP, and progressive bone destruction [22, 23]. Consistent with these findings, the peri-implant health group in the present study exhibited low inflammatory parameters, whereas the peri-implant mucositis group demonstrated marked increases in PI, GI, and BOP accompanied by only limited increases in PD. Despite these inflammatory changes, FD values did not differ significantly between peri-implant health and mucositis. This finding suggests that trabecular microarchitectural alterations may not yet be sufficiently developed during the early inflammatory stage of disease. In contrast, the peri-implantitis group exhibited the highest PD, PI, GI, and BOP values together with the lowest FD levels. These findings support the association between increasing inflammatory burden and trabecular deterioration. In addition, total FD values showed weak but statistically significant negative correlations with PD and BOP.

From a clinical perspective, the practical utility of FD analysis lies in its potential to provide a quantitative and objective surrogate for trabecular bone microarchitecture that complements conventional diagnostic methods. While standard clinical parameters such as PD and BOP remain the primary indicators of peri-implant health, FD analysis offers an incremental value by providing a mathematical assessment of the internal bone complexity that may not be fully captured during visual radiographic inspection. In practice, this method can be utilized as a diagnostic adjunct to support the clinical evaluation of peri-implant disease severity. Our findings, supported by ROC analysis, demonstrate that FD values show a moderate diagnostic performance in distinguishing peri-implantitis from healthy sites, with a notable reduction in structural complexity as the disease progresses to its destructive phase. Therefore, FD analysis can serve as an objective radiographic marker that assists clinicians in characterizing bone quality alterations, potentially providing a more comprehensive appraisal of the tissue-level changes associated with peri-implantitis.

Previous studies evaluating FD in peri-implant and periodontal conditions have reported variable and sometimes inconsistent findings [12, 14, 24–31]. Lang et al. [14] found no significant differences in FD values among peri-implant health, mucositis, and peri-implantitis groups and did not observe significant correlations between FD and clinical parameters. The authors attributed these findings to factors such as sample imbalance and small ROI dimensions. In contrast, several periodontal studies demonstrated lower FD values in diseased bone compared with healthy sites, suggesting reduced trabecular complexity in association with inflammatory bone destruction [27–31].

Studies evaluating peri-implant bone remodeling have also reported dynamic FD changes during healing and loading periods. Sansare et al. [25] observed increased FD values after implant placement, whereas Soylu et al. [27] demonstrated an initial decrease followed by gradual increases during healing. Similarly, Zeytinoğlu et al. [12] reported reduced FD values during the early loading period, while İlhan et al. [26] found no significant differences between peri-implant bone and healthy contralateral bone at 12 months. Collectively, these findings suggest that FD values may reflect time-dependent alterations in trabecular bone organization associated with inflammatory destruction, healing, and bone remodeling processes.

FD measurements may be influenced by several methodological factors, including ROI selection, imaging modality, voxel size, and image acquisition parameters [32–37]. Previous studies have demonstrated significant differences between FD values obtained from panoramic radiographs and cone-beam computed tomography (CBCT) images, suggesting that fractal outcomes may be affected not only by biological variations but also by image acquisition and reconstruction parameters [34, 35]. In particular, voxel size and reconstruction thickness may influence trabecular representation and FD calculations [35, 36]. Although CBCT provides volumetric data, its reliability for detailed trabecular microstructural evaluation remains controversial [37]. Therefore, panoramic-derived FD values should be interpreted as modality-dependent indicators of trabecular complexity rather than direct three-dimensional measurements. Another important methodological consideration involves ROI selection, as ROI location may exert a greater influence on FD values than ROI size itself [38]. In the present study, ROIs were selected parallel to the implant long axis to include the widest possible trabecular bone area adjacent to the implant surface while avoiding overlap and adjacent anatomical structures that could affect FD measurements. Nevertheless, despite efforts toward methodological standardization, anatomical variability among patients and differences in implant angulation may still have influenced ROI selection and FD measurements.

This study has several limitations. Panoramic radiographs provide lower structural detail than periapical radiographs or CBCT and are inherently susceptible to superimposition, projection, and magnification errors. In addition, the cross-sectional design prevented longitudinal evaluation of FD changes during disease progression. Because of the retrospective design and anonymized records, implant-related variables such as implant system characteristics and surface properties could not be fully evaluated. Therefore, FD findings should be interpreted cautiously in clinical practice and always together with established clinical and radiographic parameters. Future longitudinal studies with larger sample sizes, standardized implant systems, and higher-resolution imaging modalities are needed to further clarify the clinical applicability of FD analysis in peri-implant disease assessment.

## Conclusion

In conclusion, the present study indicates that FD analysis may be a useful radiographic tool for detecting trabecular bone deterioration in peri-implantitis, although its discriminatory capacity appears limited in the early stages of mucositis. In this context, FD analysis may be considered a supportive approach for the assessment of peri-implant diseases when used in conjunction with established clinical parameters. Prospective studies with larger sample sizes and the incorporation of three-dimensional imaging modalities are essential to further validate and expand these findings.

## Data Availability

No datasets were generated or analysed during the current study.

## Abbreviations

FD: Fractal Dimension
PI: Plaque Index
GI: Gingival Index
BOP: Bleeding on Probing
PD: Probing Depth
ROI: Region of Interest
